# Assessing Awareness of College Student Startup Entrepreneurs Toward Mass Entrepreneurship and Innovation From the Perspective of Educational Psychology

**DOI:** 10.3389/fpsyg.2021.690690

**Published:** 2021-08-12

**Authors:** Mingji Liu, Xingyang Yu

**Affiliations:** ^1^School of Economics and Management, Harbin University of Science and Technology, Harbin, China; ^2^School of Public Finance and Administration, Harbin University of Commerce, Harbin, China

**Keywords:** educational psychology, awareness of innovation and entrepreneurship, college students, sampling survey, startup entrepreneur

## Abstract

Against the background of economic globalization, the awareness of college student startup entrepreneurs toward mass entrepreneurship and innovation is analyzed from the perspective of educational psychology, thus responding to national development strategies. First, the *status quo* of innovation and entrepreneurship education is understood by literature analysis. Second, the existing innovation and entrepreneurship education in colleges and universities is investigated with a questionnaire survey (QS) and interviews to discover the challenges that college students face during innovation and entrepreneurship. The QS results provide a data basis for subsequent strategies. The results demonstrate that 19.86% of the students have a complete understanding of innovation and entrepreneurship, while only 9.93% of the students are interested in innovation and entrepreneurship activities. The technical significance of innovation and entrepreneurship activities only accounts for 41.84%. Moreover, the vast majority of students (79.67%) believe that the curriculum of innovation and entrepreneurship education is single, simple, and irrelevant to their majors. Meanwhile, some problems have been found: for example, the teaching faculty is insufficient, and there is not a sound entrepreneurial atmosphere. As per the survey results, an innovation and entrepreneurship education strategy for core stakeholders, namely, universities, society, government, university teachers, and students, is formulated to promote the sound development of innovation and entrepreneurship education in colleges and universities. Besides, a training strategy in line with the awareness of college students toward mass entrepreneurship and innovation is formulated based on the current situation of innovation and entrepreneurship education, which can serve as a reference and has practical significance for enriching and perfecting the innovation and entrepreneurship education system for college students.

## Introduction

As economic globalization[0mm][-4mm] accelerates, the number of university graduates increases yearly, resulting in severe employment situations and difficulties in self-employment. Data suggest that the number of college graduates in China has increased from about 5 million in 2010 to nearly 9 million in 2019, with only about 70% employment rate (Kong and Jiang, 2011; Jena, 2020). Therefore, cultivation and improvement of the comprehensive entrepreneurial quality of college students are urgently needed. With the proposal of the mass entrepreneurship and innovation initiative, China focuses on entrepreneurship activities and education while putting forward many policy packages. Thus, university entrepreneurship education has received a widespread attention.

Entrepreneurship education, as a practical course, aims to cultivate the innovative awareness, innovative spirit, and innovative ability of college students, and is designed with reasonable objectives and regularity. Additionally, higher education in China has developed rapidly in the past few years, and under the background of the mass entrepreneurship and innovation initiative, entrepreneurship education has received a great attention from the whole society. Hence, innovative talent training is guiding and innovating talent training modes of colleges and universities (Hu et al., 2018). The vigorous development of entrepreneurial activities promotes the systematization of basic entrepreneurial education theories, reformation of research methods, expansion of research scope, and improvement of practical effects. Comprehensive research on disciplines significantly influences the theory and practice of entrepreneurship education (Yan et al., 2018). Educational psychology can combine psychology and pedagogy, which studies the dynamic psychological activities of students and teachers during learning and teaching, as well as their interaction relationships (Wang et al., 2019; Chen et al., 2020). Under the background of mass entrepreneurship and innovation, teachers can understand the psychology and learning interests of students in entrepreneurship classes through educational psychology, discover learning problems of students, and grasp the learning status of students. Meanwhile, psychological dilemmas of students in learning can be revealed, which will help teachers fully understand the acceptance of entrepreneurial knowledge by students and formulate targeted teaching plans.

To sum up, while the economic development model is transforming, independent innovation must be placed in a prominent position, which will be the internal driving force of the economic development of China and the trend of sustainable development. Here are the innovation points: first, the literature analysis method is chosen to understand domestic and international innovation and entrepreneurship education. Meanwhile, from the perspective of educational psychology, the QS (questionnaire survey) and interview method are selected to investigate college students, thereby determining existing problems of college students in the process of innovation and entrepreneurship, formulating the corresponding strategies. The results provide theoretical support for the development of innovation and entrepreneurship education in colleges and universities.

## Recent Study

Under the trend of mass entrepreneurship and innovation, many colleges and universities have included courses of innovation and entrepreneurship education. The theoretical research and practical activities of innovation and entrepreneurship education in colleges and universities are highly active, and significant results have been obtained.

To contextualize entrepreneurial behaviors, Morales et al. (2019) assessed the cultural background of individual entrepreneurial values through the mastery and egalitarian cultural dimensions of Schwartz. Personal values were more important to explain entrepreneurship, and egalitarianism reduced the impact of self-improvement and openness to change values. Lang and Liu (2019) provided empirical evidence for the research on entrepreneurial motivation theory in a specific fashion entrepreneurial context by online open questionnaire collection. The results pointed out the development direction of fashion entrepreneurship courses from the perspective of college students in fashion majors. Horst and Hitters (2020) understood the impact of entrepreneurial strategies on entrepreneurial identity development and entrepreneurial knowledge construction. They found that entrepreneurial knowledge construction helped people understand strategies in the early stage of entrepreneurship. Sun et al. (2020) analyzed online innovation and entrepreneurship education in China and discussed implementation strategies, namely, curriculum setting, teaching method reform, faculty construction, teaching content, and Internet innovation and entrepreneurship practice. Boubker et al. (2021) analyzed the influence of entrepreneurship education on the entrepreneurial intention of Moroccan students and proposed that entrepreneurial intention depended on four variables: entrepreneurship education, entrepreneurial attitude, perceived social norms, and perceived entrepreneurial ability. Meanwhile, the research results indicated that there was a significant statistical relationship between entrepreneurship education, entrepreneurial attitude, and entrepreneurial intention of management students.

The above studies reveal that innovation and entrepreneurship are activities with characteristics of the times. Innovation and entrepreneurship education have become trendy educational topics. Developing innovation and entrepreneurship education has also become a focus and resonance of higher education in the world. However, the current research is relatively shallow, non-systematic, and incomplete, which singles out the innovation and entrepreneurship education of colleges and universities from professional courses. Therefore, the awareness of innovation and entrepreneurship of college students is understood from the perspective of educational psychology. Then, corresponding strategies are proposed, which are of great significance to promoting entrepreneurial awareness of college students.

## Innovation and Entrepreneurship Status of College Students

Under the background of economic transformation, the innovation-driven organizational mechanism is discussed. On this basis, the requirements for innovation-driven entrepreneurship education in colleges and universities are analyzed, and the corresponding strategies are explored through case analysis.

### Analysis Method

#### Research Purpose and Significance

Theoretically, the purposes are to enrich the theoretical system of innovation and entrepreneurship education in China, put forward systematic and innovative suggestions for innovation and entrepreneurship education courses in colleges and universities, and provide theoretical support to meet the needs of innovation-driven entrepreneurship education in Chinese universities.

Practically, the purposes are to provide scientific guidance for the practice of innovation and entrepreneurship education in colleges and universities and help outstanding college students recognize innovation and entrepreneurship education, bravely innovate and start businesses, apply what they have learned, and develop an adaptable innovative and entrepreneurial thinking. Simultaneously, college students are instructed to carry out system innovation to help innovation and entrepreneurship education better interact and promote each other with the regional socio-economic environment (Rosca et al., 2020). Therefore, the research on innovation and entrepreneurship education for college students is very important.

#### Research Methodology

**Figure 1:**
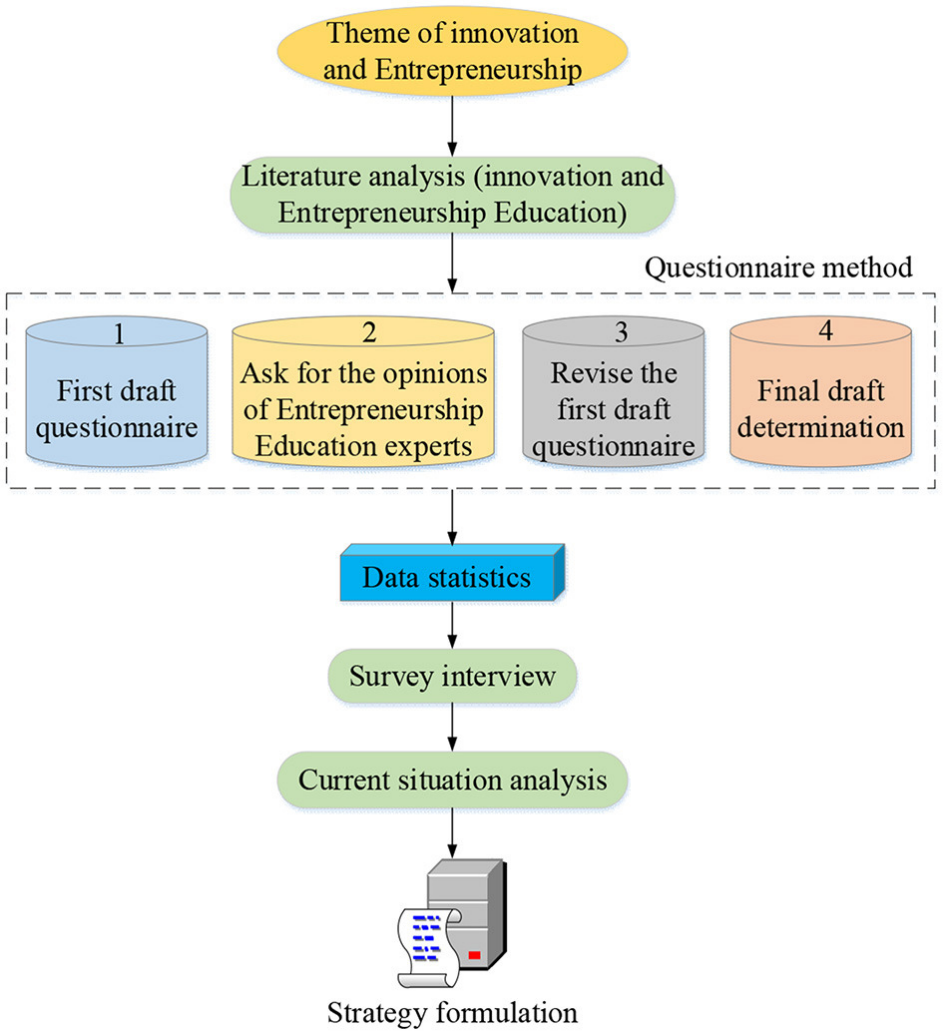
Analysis of the *status quo* of innovation and entrepreneurship of college students and flowchart of strategy formulation.

The *status quo* of the understanding of innovation and entrepreneurship of college students is analyzed by literature analysis (Bendell et al., 2019), QS (Lee et al., 2018), and field interviews (Yadav and Goyal, 2015). The specific analysis process and strategy formulation are shown in Figure 1.

First, the literature analysis method: specifically, the views of domestic and foreign education thoughts and schools as well as the current research materials are comprehensively utilized, and the situation of innovation and entrepreneurship education is analyzed from the perspective of value. Second, the QS method: the investigation and analysis of college students reveal the actual problems of innovation and entrepreneurship practices of college students, thereby providing a data basis for strategy formulation. Additionally, an interview survey is conducted to confirm the results of the QS. Finally, the failure factors of innovation and entrepreneurship of college students are explored through interdisciplinary research methods, thus providing a theoretical basis for strategy formulation.

### Impact of Educational Psychology on Awareness of College Students Toward Mass Entrepreneurship and Innovation

Psychologists believe that many external and internal factors stimulate the behaviors of individuals by satisfying their needs. Motivation is the internal driving force of entrepreneurial activities (Rokhman and Ahamed, 2015). Educational psychology is closely associated with general psychology and pedagogy; however, it holds different opinions on research objects. First, it researches educational methods for the comprehensive development of morality, intelligence, and physique according to the pedagogy systems. Second, educational psychology studies the human psychological structure and determines its theoretical system as per the laws of psychological activities in the process. Third, the foremost task of educational psychology is to study the nature, conditions, effects, and evaluation of classroom learning. Educational psychology should focus on the study of learning theory, especially the learning theory of acceptance of knowledge and skills by the students. Fourth, educational psychology probes into various psychological phenomena and development laws in educational and teaching activities, thereby promoting education and teaching and achieving the optimal development of students (Fiore et al., 2019; Zreen et al., 2019; Boldureanu et al., 2020; Ratten and Jones, 2021).

Therefore, the psychology and ideas of college student startup entrepreneurs are developed and cultivated to change their cognitions and promote the transformation of their thinking and value orientations. While students are encouraged for entrepreneurship, they are educated to produce healthy and positive entrepreneurial attitudes and behavioral tendencies, thereby benefiting both individual and social developments. This is important for guiding college students to establish correct entrepreneurial values and promoting the sound development of the entrepreneurial ecosystem.

### Investigation and Analysis of Entrepreneurship and Innovation Status of College Students

#### QS Design

According to the above analysis, the knowledge, attitude, and practices (KAP) questionnaire (Kier and McMullen, 2018) is applied for the empirical investigation on the awareness of innovation and entrepreneurship of college students, considering the studies of scholars in relevant fields. The questionnaire is designed to investigate the awareness of innovation and entrepreneurship of college students, the technical significance of innovative activities, and the awareness of innovation and entrepreneurship education of students in their schools (Danish et al., 2019; Elliott et al., 2020; Winkler et al., 2021). The current situation, issues, and influencing factors of innovative and entrepreneurial values of college students can be understood through the QS. Training strategies that are both common and regional can be proposed to help college students cultivate innovative and entrepreneurial values. The QS is designed with three steps. First, literature and data are checked. Following the literature on countermeasures for cultivating the entrepreneurial values of college students worldwide, data are sorted and collected from different channels before analysis. To ensure the validity of the QS, relevant variables involved are preliminarily determined. Second, the first draft QS is designed. Based on the literature review and data processing, the QS is initially designed. Then, the rationality of the indicator design is solicited from experts and scholars in the academic field. The first draft of the QS is determined after relevant revisions based on their opinions. Finally, the first draft of the QS is revised. The QS adopts a five-point Likert scale (Palmer et al., 2019), and the results are set as completely agree (5 points), agree (4 points), uncertain (3 points), disagree (2 points), and strongly disagree (1 point). Importantly, the processes of questionnaire design, distribution, and data collection do not involve any personal privacy. The entire survey process is conducted with the consent of the participants (no <18 years old). This QS is not open to the public and is only for academic purposes.

#### Survey Authenticity Analysis

The authenticity of the survey is analyzed from three perspectives: professionalism of the survey, randomness of the data, and scientific nature of the data. The first perspective is survey professionalism. Some third-party institutions or college student volunteers are invited to participate in the survey. These third-party participants are screened and trained in advance. They are aware of the survey precautions and survey significance, ensuring that they are serious and responsible to guarantee data authenticity. The second perspective is data randomness. To enhance the rigor of this survey, three representative local undergraduate colleges and universities are selected where the equivalent QS is issued for the sampling survey. The time for answering the questions is controlled within 15 min. This QS is distributed on-site, and survey interviews will be conducted according to the QS results, thereby ensuring its effectiveness. The third perspective is the scientific nature of the data. After the investigation, the collected information will be sorted and analyzed to remove useless data. Then, the screened data are integrated and summarized to reflect the current problems intuitively. Some suggestions are put forward to provide a guarantee for formulating scientific and reasonable strategies. Reliability verification is performed to determine the validity of the data. Methods involving statistics, psychology, and other disciplines are combined to ensure the scientific nature of the data.

#### Survey Data Statistics

A quality inspection of the QS is required to verify the reliability and stability of the collected data and the indicator system about the awareness of innovation and entrepreneurship of college students. The reliability coefficient used is Cronbach α (Akhter et al., 2020), which verifies the internal consistency between the scores of each question in the QS. SPSS 24.0 is chosen for α reliability coefficient and KMO value evaluation, as shown in Table 1.

**Table 1:** Reliability and validity of the formal survey scale.

**Variable**	**Variable α coefficient**	**KMO value**	**Chi-square value**	**Sig**
Awareness of innovation and entrepreneurship	0.839	0.836	532.918	0.000
Innovative activities' technical significance	0.857	0.874	467.153	0.000
Awareness of innovation and entrepreneurship education	0.862	0.891	1061.079	0.000

Table 1 displays that Cronbach's α coefficient of each variable is larger than 0.8, KMO >0.7, and Sig = 0 and < 0.05, proving that the designed QS has strong internal consistency, stability, and high reliability. The reliability and validity of each scale are in line with the specific norms of case analysis, meeting the requirements of factor analysis. Furthermore, AMOS 24.0 is chosen to test the fitting indicator of each variable scale to verify the relationship between the factors, as shown in Table 2.

**Table 2:** Confirmatory factor analysis (CFA) fitting indicator table of the formal QS scale.

**Variable**	**Awareness of innovation and entrepreneurship**	**Innovative activities' technical significance**	**Awareness of innovation and entrepreneurship education**
Average factor load (standardized regression coefficient)	0.760	0.751	0.764
CR value	0.510	0.572	0.561
AVE variance	0.840	0.840	0.910
χ^2^/df	3.332	2.637	1.792
RMSEA	0.086	0.072	0.051
GFI	0.971	0.993	0.975
NFI	0.976	0.985	0.978
CFI	0.979	0.994	0.991
IFI	0.979	0.994	0.991

Table 2 displays the fitting indicator CFA of each variable, showing that, for awareness of innovation and entrepreneurship, the average factor load is 0.76, the CR value is 0.51, and the AVE variance is 0.84. In terms of the technical significance of innovative activities, the average factor load is 0.751, the CR value is 0.572, and the AVE variance is 0.84. The average factor load of awareness of innovation and entrepreneurship education is 0.764, the CR value is 0.561, the AVE variance is 0.91, and the GFI, CFI, and NFI of each dimension variable are >0.9, while the RMSEA is <0.1. Thus, the fitting indicator of the model is good, and the model is verified. Therefore, the QS is reasonable and effective, and can provide a basis for further research.

Three local undergraduate colleges and universities are selected, where equivalent questionnaires are issued for the sampling survey to reduce the deviation and enhance the rigor of the survey results and the scientificity and representativeness of this survey. Four hundred and fifty QS are distributed, of which 436 copies are returned, with a response rate of 96.89%, indicating that this QS is effective. Furthermore, to ensure the validity of the QS, the 436 valid QSs are carefully reviewed, and problematic QSs are eliminated. At the same time, to reduce the error of the QS and ensure its even distribution in the three different undergraduate colleges, 141 valid QSs are selected from each school, totaling to 423 valid QSs.

## Results and Discussion

The 423 QSs are analyzed, and the results are summarized in Table 3. Most of the survey objects are women, accounting for 52.48%. Nevertheless, the ratio of men to women tends to be balanced on the whole. In terms of grade distribution, the survey objects are first-, second-, and third-year students of colleges and universities, with fewer fourth-year students. A possible reason is that graduates are busy looking for jobs, preparing for postgraduate exams, and applying for opportunities to study abroad; hence, they cannot participate in the survey. In terms of professional distribution, college students majoring in Humanities and Social Sciences, Science and Engineering, and Economic Management account for 35.7, 34.04, and 23.88%, respectively, occupying the vast majority of the survey objects. College students majoring in Sports, Art, Agriculture, and Medicine account for a tiny proportion, which may be caused by the nature and professional settings of the colleges and universities surveyed.

**Table 3:** Basic situation of the survey objects.

		**Statistics**	**Percentage (%)**
Gender	Male	201	47.52
	Female	222	52.48
Grade	First-year in university	177	41.84
	Second-year in university	130	30.73
	Third-year in university	71	16.78
	Fourth-year in university	45	10.64
Disciplines	Economic management	101	23.88
	Science and engineering	144	34.04
	Humanities and social sciences	151	35.70
	Sports and art	17	4.02
	Agriculture and medicine	10	23.64

### Results and Analysis of QS

#### Investigation Results of Awareness of Students Toward Innovation and Entrepreneurship

**Figure 2:**
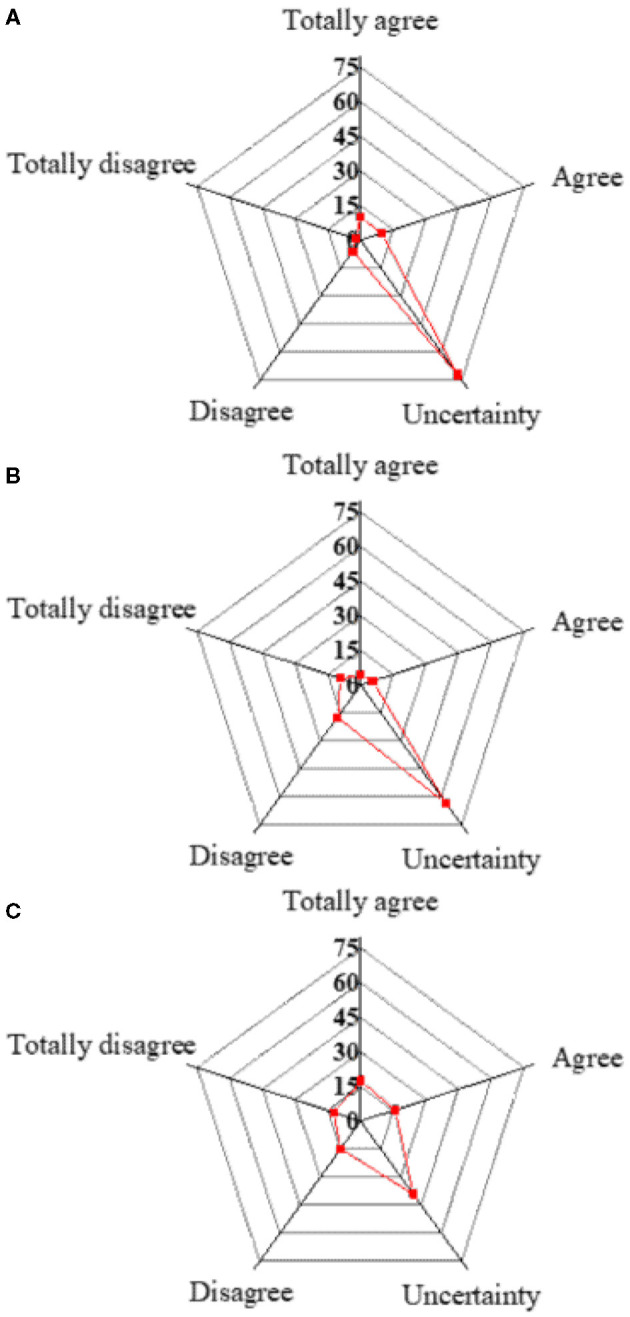
Survey results of innovation and entrepreneurship of college students. **(A)** Having a complete understanding of innovation and entrepreneurship education. **(B)** Being interested in various innovation and entrepreneurship activities. **(C)** Knowing about innovation and entrepreneurship policies.

Data of the sampling survey about innovative awareness of college students are analyzed, and the results are presented in Figure 2. Only 19.86% of the students have a complete understanding of the innovation and entrepreneurship concept, and 80.14% believe that it is unnecessary to have a complete understanding of innovation and entrepreneurship education. Only 9.93% are interested in various innovative and entrepreneurial activities, such as the Challenge Cup, while the proportion of those uninterested in these activities reaches 90%. Moreover, 33.57% of the students know about the policies associated with innovation and entrepreneurship, and 66.43% are not interested in understanding such policies.

#### Survey Results of the Technical Significance of Innovation and Entrepreneurship Activities

**Figure 3:**
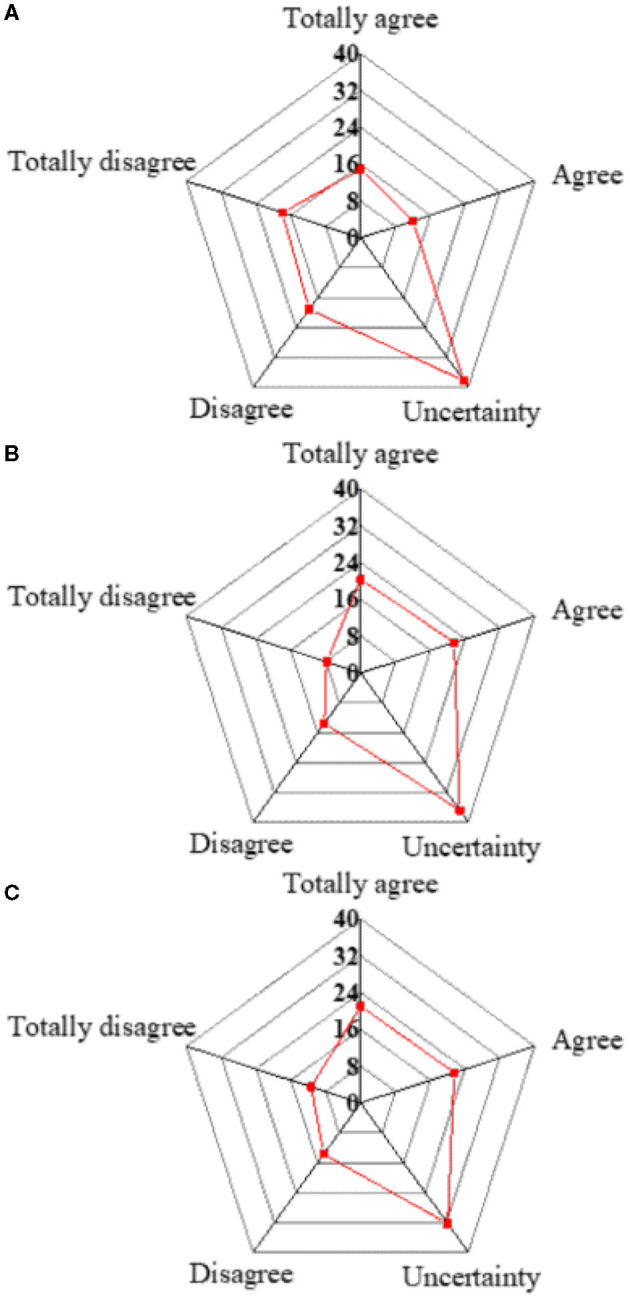
Survey results of the technical significance of innovation and entrepreneurship activities. **(A)** Understanding the “Challenge Cup” Innovation and Entrepreneurship Competition. **(B)** Entries submitted have technical significance. **(C)** Entries submitted are associated with professional knowledge of students.

Survey results about the technical significance of innovation and entrepreneurship activities are presented in Figure 3. Students who understand the Challenge Cup Innovation and Entrepreneurship Competition account for 26.95%, while those who agree that the entries have technical significance and are connected with their professional knowledge account for 41.84 and 42.55%, respectively. The interviews suggest that most award-winning studies are finished under the instructions of teachers. Except for national or provincial competitions, colleges and universities have organized very few innovation activities. Few students have participated in the innovation activities held as well.

#### Survey Results of Innovation and Entrepreneurship Education

The development of innovation and entrepreneurship education in colleges and universities is surveyed, and the results are analyzed as follows.

**Figure 4:**
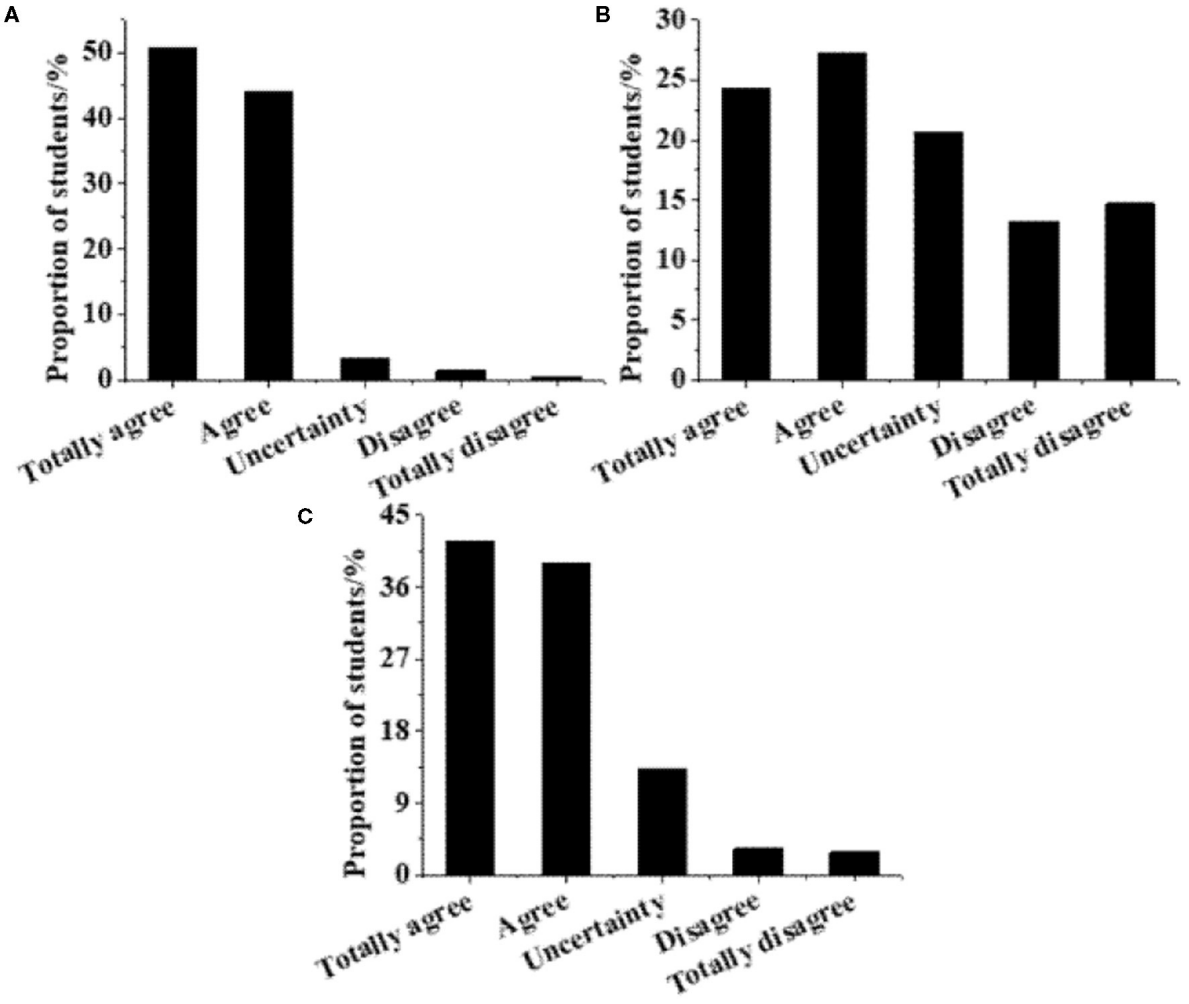
Survey results of understanding of innovation and entrepreneurship education of college students. **(A)** Innovation and entrepreneurship education is to solve employment difficulties. **(B)** Innovation and entrepreneurship education is extracurricular activities and competitions. **(C)** Innovation and entrepreneurship education is conventional skills guidance.

**Figure 5:**
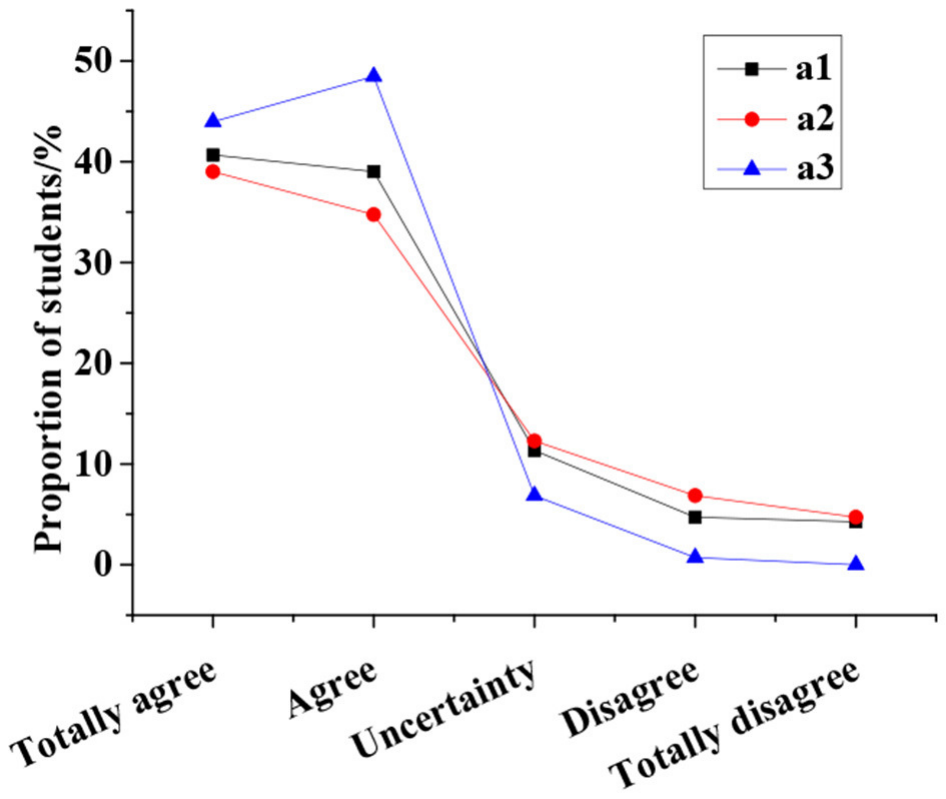
Survey results of the current status of the innovation and entrepreneurship education system (a1. Single course, simple content, and irrelevant to majors; a2. Insufficient teachers and low level; a3. Courses are optional or examination ones).

The understanding of innovation and entrepreneurship education of college students is surveyed, and the results are displayed in Figure 4. The vast majority of students believe that innovation and entrepreneurship education solve employment difficulties (94.8%). The proportion of students who believe that innovation and entrepreneurship education are extracurricular activities and conventional skill guidance account for 51.54 and 80.62%, respectively. Furthermore, the current systems of innovation and entrepreneurship education are analyzed, and the results are summarized in Figure 5. Specifically, 79.67% of the students think that the curriculum is single, simple, and irrelevant to their majors; 73.76% think that the teachers are insufficient and their teaching skills are poor; 92.43% of the students think that the innovation and entrepreneurship courses are elective or examinations.

**Figure 6:**
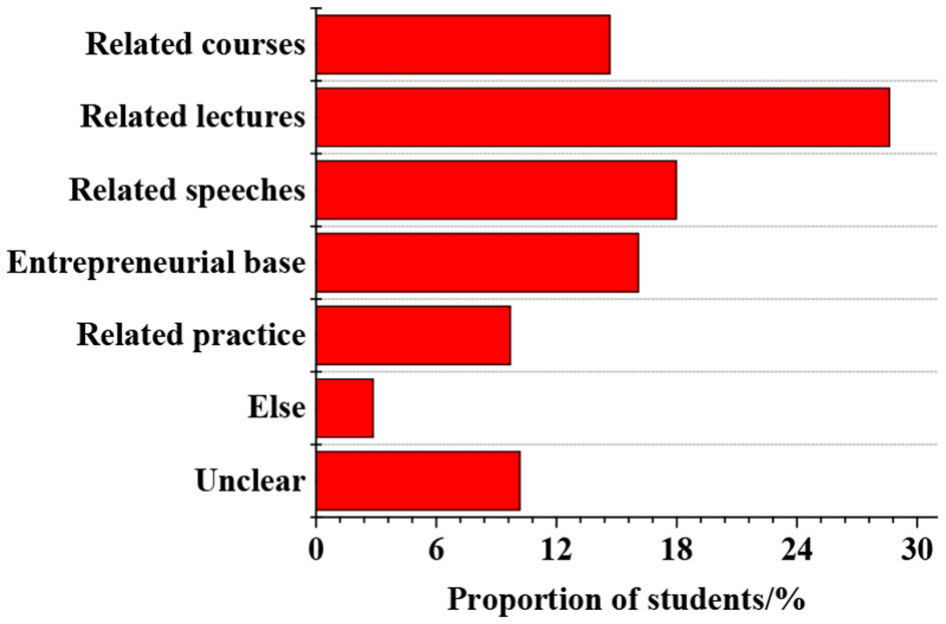
Survey results of innovation and entrepreneurship activities held by colleges and universities.

**Figure 7:**
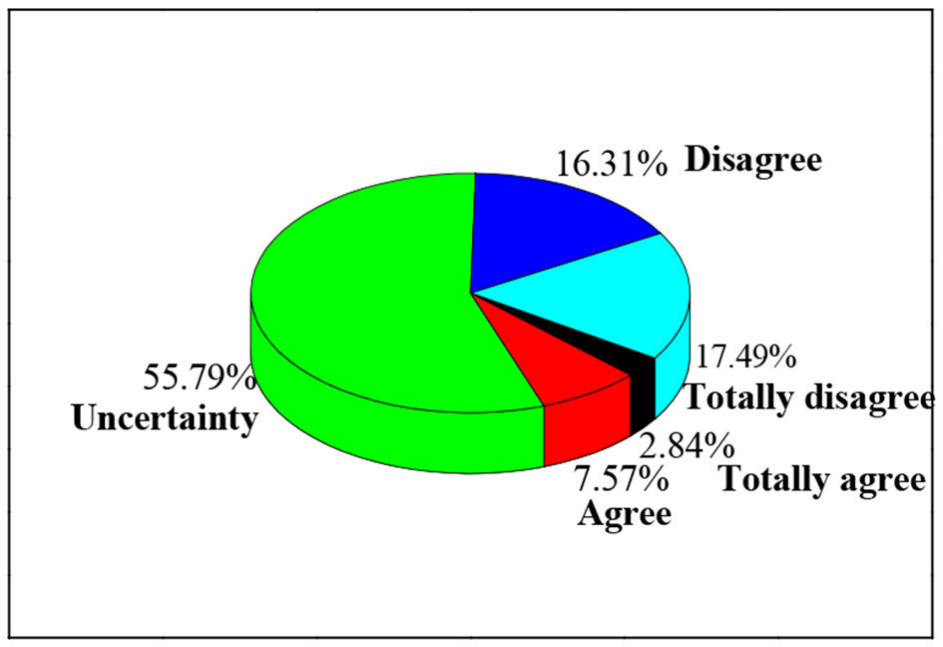
Survey results of interests in innovation and entrepreneurship of college students.

The *status quo* of innovation and entrepreneurship activities held by colleges and universities is surveyed, as shown in Figure 6. The results suggest that 28.61% of college students think that the main forms of innovation and entrepreneurship activities held by colleges and universities are lectures. Other activities include speeches (17.97%), entrepreneurship bases (16.08%), and courses (14.66%). Moreover, 2.84% of the college students report other forms, and 10.17% do not know any activities. In general, 14.66% of the college students believe that the main forms of innovation and entrepreneurship activities held by colleges and universities are teaching activities, while 85.34% report non-teaching activities. Furthermore, the entrepreneurial atmosphere of college students is investigated, as shown in Figure 7. Students who are uncertain account for 55.79%, the largest proportion, followed by those who agree that there is not a strong atmosphere, accounting for 17.49%. Students who agree that the entrepreneurial atmosphere is strong and very strong account for <10%.

### Multiple Regression Analysis of Each Dimension

The comprehensive mechanism of various factors in the entrepreneurship education system is complex. Here, the three dimensions of the student QS and satisfaction are statistically analyzed by regression analysis. The result shows that the three dimensions have a significant impact on the satisfaction of students. Meanwhile, multiple regression analysis is conducted to test the relationship between the five dimensions and satisfaction. The result indicates that only the dimension of innovation and entrepreneurship education has a significant relationship with satisfaction, specifically, β = 0.518, *p* < 0.001. Thus, the better the innovation and entrepreneurship education, the higher the satisfaction, and there is a significant positive correlation between the satisfaction and innovation of students and entrepreneurship education. By comparison, the awareness of innovation and entrepreneurship (β = 0.014, *p* = 0.611) and the technical significance of innovative activities (β = −0.021, *p* = 0.635) have no significant relationship with the satisfaction of student. The results are shown in Table 4.

**Table 4:** Multiple regression analysis of each dimension.

	**Satisfaction**		
	***B***	***SE B***	**β**
Awareness of innovation and entrepreneurship	0.006	0.015	0.014
Innovative activities' technical significance	−0.007	0.016	−0.021
Innovation and entrepreneurship education	0.154^***^	0.015	0.518^***^
*R* ^2^	0.502		
*Adj R* ^2^	0.489		
*F*	111.047^***^		
*df*	(5,451)		

*Annotation: N = 555;*

*** *p < 0.001*.

### Discussion of Survey Results

Awareness of innovation and entrepreneurship refers to desires and thoughts of people to create or explore ideas or things that they have never felt or have a deep interest in. It shows the will, aspirations, interests, reveries, attitudes, ideas, and emotions of people in creative activities, which is an aggressive, positive, and extremely fulfilling form of expressing human thinking and awareness activities. Besides, it is the premise of human creativity and, ultimately, meets needs of people for social and personal development (Almahry et al., 2018; Briegas et al., 2020). Figure 2 illustrates that awareness of innovation of college students is weak, and that their cognition of innovation and entrepreneurship is biased. Besides, they are not interested in innovation and entrepreneurship activities and are not motivated to participate, with very few numbers of self-employed entrepreneurs. The above situation cannot meet the requirements of the times; however, they are precisely the key factors in training innovative talents. The innovation and entrepreneurship activities of colleges and universities should be based on majors and take professional knowledge, invention, and innovation as cores to lay a good foundation for knowledge-based entrepreneurship.

In terms of course understanding, most students do not take innovation and entrepreneurship education seriously, lack a scientific learning mechanism, and have a biased cognition; worse, they regard innovation and entrepreneurship education simply as a utilitarian activity (Garbuio et al., 2018). In terms of course systematicness, the feedback of the students suggests that the innovation and entrepreneurship education courses in colleges and universities are too simple, disintegrated, less correlated with professional education and have not formed a scientific and systematic discipline. Besides, the teaching faculty lacks professional skills and experiences. These are well-reflected in the investigation of Washington et al. (2020). Many surveys imply that almost all efforts have been put on the enhancement of key factors of entrepreneurship education in the past. Apparently, to solve existing problems and reform traditional entrepreneurship education, key factors should be paid great attention to. At the same time, emphases should be put on the integrity, openness, systematicness, and balance of entrepreneurship education. Particularly, without systematic planning, comprehensive perspective, or in-depth thinking on the structure and elements, and their relationship, the effect of entrepreneurship education will be greatly reduced. In short, most colleges and universities have not yet formed a systematic innovation and entrepreneurship education system, and the existing course is alienated from subject education and the overall teaching system.

### Strategies for Innovation and Entrepreneurship Education

A successful education model should be formed based on the cooperation of relevant subjects, especially for the construction and improvement of entrepreneurship education. It is necessary to closely integrate core stakeholders, namely, universities, society, government, university administrators, university teachers, and students. Enhancement in exchanges and interactions of all parties helps effectively utilize resources, improve the effectiveness of the entrepreneurship education model, and promote the sound development of innovation and entrepreneurship education in colleges and universities. Hence, the following strategies are proposed based on the investigation and analysis of the awareness of innovation and entrepreneurship of college students and the *status quo* of innovation and entrepreneurship education.

First, the role of the government in the entrepreneurial education model of colleges and universities should be clarified. The government provides necessary support by improving the laws and building a cooperation platform. Second, a comprehensive social support system for entrepreneurship education should be established. Under reinforced multi-party cooperation between universities and enterprises, the social entrepreneurial environment can be optimized, so that off-campus funds can be introduced to support the development of entrepreneurial activities in colleges and universities. Also, the needs of enterprises can be directly connected to innovation and entrepreneurship projects of universities, accelerating the concretization of outstanding projects of entrepreneurial education in colleges and universities. Third, the overall construction of entrepreneurial education systems of colleges and universities should be accelerated. Colleges and universities can improve the strength and quantity of teachers responsible for entrepreneurship education by constructing an entrepreneurship education curriculum system, and, simultaneously, further improve the construction of entrepreneurship incubation platforms. Fourth, the quality and ability of teachers responsible for entrepreneurship education should be improved. Besides grasping the essence of entrepreneurship education, teachers should enhance their teaching adaptability, probe into entrepreneurship education, and promote the development of theoretical and practical teaching. Fifth, the enthusiasm of students to participate in entrepreneurship education should be mobilized. Targeted help can be provided based on a mastery of the needs of students. Additionally, publicity can be performed *via* multiple channels to promote entrepreneurship education (Galvão et al., 2020; Mei and Symaco, 2020).

## Conclusion

The awareness of new venture entrepreneurs among college students toward mass entrepreneurship and innovation is analyzed from the perspective of educational psychology. The understanding of entrepreneurship education of contemporary college students is investigated by literature analysis, QS, and interviews. The results suggest that contemporary college students have weak awareness of innovation and entrepreneurship. Some students (41.84%) believe that innovation and entrepreneurship activities do not have technical significance, with a single curriculum that is irrelevant to the majors of students. Finally, strategies are formulated for core stakeholders, namely, universities, society, government, university teachers, and students, to provide a reference for the sound development of innovation and entrepreneurship education in colleges and universities. However, there are also some shortcomings. First, the scope of the survey and the scale of the sample need further expansion. College students are mainly studied here, but core stakeholders, such as universities, society, government, university administrators, university teachers, and students all are involved in the actual innovation and entrepreneurship education. Therefore, in the follow-up, subject universality should be extended, and more abundant analysis methods can be adopted to compare and analyze the situation of different survey subjects. Meanwhile, the influence of subjective factors should be minimized in QS design, the QS model should be optimized, and the results variables should be introduced for further analysis.

## Data Availability Statement

The raw data supporting the conclusions of this article will be made available by the authors, without undue reservation.

## Ethics Statement

The studies involving human participants were reviewed and approved by Harbin University of Science and Technology Ethics Committee. The patients/participants provided their written informed consent to participate in this study. Written informed consent was obtained from the individual(s) for the publication of any potentially identifiable images or data included in this article.

## Author Contributions

All authors listed have made a substantial, direct and intellectual contribution to the work, and approved it for publication.

## Conflict of Interest

The authors declare that the research was conducted in the absence of any commercial or financial relationships that could be construed as a potential conflict of interest.

## Publisher's Note

All claims expressed in this article are solely those of the authors and do not necessarily represent those of their affiliated organizations, or those of the publisher, the editors and the reviewers. Any product that may be evaluated in this article, or claim that may be made by its manufacturer, is not guaranteed or endorsed by the publisher.
