# Supplementary material for: Assessing Awareness of College Student Startup Entrepreneurs Toward Mass Entrepreneurship and Innovation From the Perspective of Educational Psychology
Source: Front Psychol. 2021 Aug 12;12:690690. doi: 10.3389/fpsyg.2021.690690 (PMC8387721; doi:10.3389/fpsyg.2021.690690)
Supplement: Supplementary file 1 [file Table_1.DOC]

1. What’s your gender？

A. Male B. Female

2. Which grade are you in？

A. Freshman B. Sohpormore C. Junior D. Senior

3. What’s your major？

A. Economics and Management B. Science and Engineering C. Humanities and Social Sciences D. Sports and Art E. Agriculture and Medicine

**Dimensions of awareness of innovation and entrepreneurship**

4. Do you think you have a complete understanding of innovation and entrepreneurship education?

A. Totally agree B. Agree C. Uncertainty D. Disagree E. Totally disagree

5. Do you think you are interested in all kinds of innovation and entrepreneurship activities?

A. Totally agree B. Agree C. Uncertainty D. Disagree E. Totally disagree

6. Do you think you understand the policies related to innovation and entrepreneurship?

A. Totally agree B. Agree C. Uncertainty D. Disagree E. Totally disagree

**Dimension of innovative activities’ technical significance**

7. Do you think you understand the Challenge Cup Innovation and Entrepreneurship Competition?

A. Totally agree B. Agree C. Uncertainty D. Disagree E. Totally disagree

8. Do you think the entry has higher technical content?

A. Totally agree B. Agree C. Uncertainty D. Disagree E. Totally disagree

9. Do you think the entry is related to the student's professional knowledge?

A. Totally agree B. Agree C. Uncertainty D. Disagree E. Totally disagree

**Dimension of innovation and entrepreneurship education**

10. Do you think innovation and entrepreneurship education is to solve employment difficulties?

A. Totally agree B. Agree C. Uncertainty D. Disagree E. Totally disagree

11. Do you think innovation and entrepreneurship education is an extracurricular activity or a competition?

A. Totally agree B. Agree C. Uncertainty D. Disagree E. Totally disagree

12. Do you think innovation and entrepreneurship education is conventional skills guidance?

A. Totally agree B. Agree C. Uncertainty D. Disagree E. Totally disagree

13. Do you think innovation and entrepreneurship education is a single course, with simple content, and irrelevant to major?

A. Totally agree B. Agree C. Uncertainty D. Disagree E. Totally disagree

14. Do you think the teachers are insufficient, and their skills are low?

A. Totally agree B. Agree C. Uncertainty D. Disagree E. Totally disagree

15. Do you think the innovation and entrepreneurship education course is an elective course or an examination course?

A. Totally agree B. Agree C. Uncertainty D. Disagree E. Totally disagree

16. What are the activities related to innovation and entrepreneurship carried out by colleges and universities?

A. Related lectures B. Related speeches C. Entrepreneurial base D. Related courses E Related practice F. Else G. Unclear

17. Do you think there is a strong atmosphere for innovation and entrepreneurship among college students?

A. Totally agree B. Agree C. Uncertainty D. Disagree E. Totally disagree
